# Measurements of Generated Energy/Electrical Quantities from Locomotion Activities Using Piezoelectric Wearable Sensors for Body Motion Energy Harvesting

**DOI:** 10.3390/s16040524

**Published:** 2016-04-12

**Authors:** Antonino Proto, Marek Penhaker, Daniele Bibbo, David Vala, Silvia Conforto, Maurizio Schmid

**Affiliations:** 1 Department of Engineering, University of Roma Tre, Via Vito Volterra, 62, Rome 00146, Italy; daniele.bibbo@uniroma3.it (D.B.); silvia.conforto@uniroma3.it (S.C.); maurizio.schmid@uniroma3.it (M.S.); 2 Department of Cybernetics and Biomedical Engineering, VSB-Technical University of Ostrava, 17 Listopadu 15, Ostrava-Poruba 70833, Czech Republic; marek.penhaker@vsb.cz (M.P.); david.vala@vsb.cz (D.V.)

**Keywords:** piezoelectric transducers, body motion energy harvesting, elastic fabric

## Abstract

In this paper, two different piezoelectric transducers—a ceramic piezoelectric, lead zirconate titanate (PZT), and a polymeric piezoelectric, polyvinylidene fluoride (PVDF)—were compared in terms of energy that could be harvested during locomotion activities. The transducers were placed into a tight suit in proximity of the main body joints. Initial testing was performed by placing the transducers on the neck, shoulder, elbow, wrist, hip, knee and ankle; then, five locomotion activities—walking, walking up and down stairs, jogging and running—were chosen for the tests. The values of the power output measured during the five activities were in the range 6 µW–74 µW using both transducers for each joint.

## 1. Introduction

Body motion energy harvesting (BMEH) means recovering energy from body movement. BMEH has been the object of study by researchers from around the world for the past twenty years.

Starner [1], who is considered one of the first researchers who studied energy harvesting from human motion, explored the possibility of recovering the energy produced by body movements, during everyday activities. He pointed out that human beings produce the highest amount of energy during walking. Thus, Shenck and Paradiso [2] developed an energy harvesting system, mounted on the shoes, that enables one to power a wide range of body-worn devices. Then, Gonzalez *et al.* [3] and Niu *et al.* [4] analyzed the feasibility of using the energy harvested from the human body to power wearable sensors, which include the functions of data processing and wireless communication [5,6].

Following the same logic, Rome *et al.* [7] developed a suspended-load backpack that converts mechanical energy of the vertical movement of the carried load into electrical energy. In addition, Donelan *et al.* [8] developed a biomechanical energy harvester mounted on the knee that provides power generation at the end of the swing phase, thus assisting deceleration of the knee joint during walking and jogging.

Mitcheson *et al.* [9] reviewed the principles in a motion-driven miniature energy harvester to introduce the basis for a wearable and comfortable body-worn energy harvesting system. Vullers *et al.* [10] published a review dealing with the techniques of micropower energy harvesting in order to confirm the theory, earlier discussed by Huang *et al.* [11] and Hanson *et al.* [12], about the wireless body sensor network (WBSN), according to which reliable energy harvesting has now become a reality for human conditions monitoring.

Nowadays, BMEH systems are becoming fundamental for sports [13], medical [14] and military [15] applications. The research trend is to develop smart clothes with incorporated BMEH systems. Stoppa and Chiolerio [16] presented a paper on recent progress in the field of smart textiles, and Misra *et al.* [17] presented a paper on flexible technologies that enable ultra-long battery lifetime and user comfort.

Despite a large body of literature focusing on the development and testing of BMEH systems, to the authors’ knowledge there is a notable lack of studies targeting the optimization of harvester kinds, and their placement, to be used in everyday life activities.

To fill this gap, in this work, piezoelectric transducers placed in a tight suit were tested and compared to find out the best location where the BMEH system can be placed, to maximize energy production in everyday life. To this end, individual body movements of single body joints were tested, and then five different locomotion activities were chosen to test the sensors in real-life conditions. The positions of the BMEH systems were chosen taking into account the wearability of the transducers and the user comfort during exercises, with the perspective of incorporating them into smart clothing.

## 2. Relevance of the Problem

BMEH is an energy harvesting subcategory that is a part of the broader category of energy harvesting from environmental vibrations. While environmental vibration energy harvesting has been extensively studied recently [9], BMEH is quite a new research field for scientists.

The mechanical energy from environmental vibrations can be converted into electrical energy by electromagnetic, electrostatic, magnetostrictive and piezoelectric transducers: in the studied literature, the electromagnetic, electrostatic and piezoelectric transducers dominate. The work presented by Roundy [18] provides a general theory that can be used to compare these different transduction approaches.

In conventional macro-scale engineering, environmental vibration transducers are mostly based on the electromagnetic technique, while in small-scale energy harvesting, electrostatic and piezoelectric transducers are more practical and better suited to microelectromechanical (MEMS) implementation [9]. The main difference between piezoelectric and electrostatic transducers is that the electrostatic transducer must be powered [19] to function, and therefore the piezoelectric transducers were chosen for our purpose.

The most common types of materials for piezoelectric transduction are lead zirconate titanate (PZT) and polyvinylidene fluoride (PVDF) [20]. Other piezoelectric materials are barium titanate (BaTiO_3_) presented by Koka *et al.* [21] and Zhang *et al.* [22], micro-fiber composites (MFC) illustrated by Kranz *et al.* [23], and active fiber composites (AFC) discussed by Dürager [24]. In addition, lead magnesium niobate-lead titanate (PMN-PT) presented by Halim *et al.* [25] and zinc oxide nanowires (ZnO-NWs) presented by Bai *et al.* [26] are materials with piezoelectric physical properties.

When comparing the PZT and PVDF transducers [27], the former is better in terms of transduction coefficients, while the latter is less expensive and more flexible, and it can add pyroelectric properties.

PZT transducers can be divided into two categories: hard PZT and soft PZT. Hard PZT are generally not suitable for BMEH because they are not flexible enough to accommodate body joint rotations. For this purpose, soft PZT and PVDF transducers were chosen for this work.

The fundamental purpose in this paper is to quantify the energy amount that can be recovered from body movements: to this end, the wearable piezoelectric transducers were incorporated into different slots within a tight body suit; the measurements of output voltage were collected during the execution of different activities, which will be specified in the following paragraph.

## 3. Materials and Methods

### 3.1. Piezoelectric Transducers Chosen

**Figure 1:**
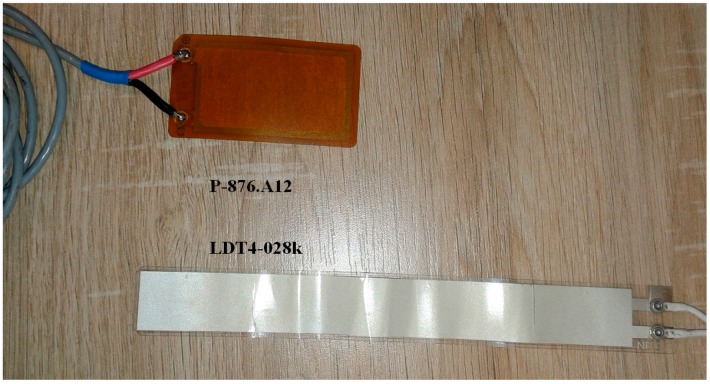
The P-876.A12 sensor [28] and LDT4-028k sensor [29].

Piezoelectric transducers convert mechanical deformations into electrical energy, *i.e.*, direct piezoelectric effect. Thanks to this property, the piezoelectric transducers can produce electrical energy from the movements of the body parts to which they adhere. The P-876.A12 sensor [28] and the LDT4-028k sensor [29] (Figure 1) were chosen to analyze the power output of a BMEH system, respectively based on soft PZT or on PVDF.

The features of both P-876.A12 and LDT4-028k are listed in Table 1.

While examining the piezoelectric transducers behavior, under conditions to approximate an open-circuit voltage, the generated voltage value from piezoelectric transducers is:

(1) $$V=\frac{t}{\varepsilon_{0}\varepsilon_{r}}d_{31}Y\frac{\Delta l}{l}$$

In applications of BMEH, it is assumed that the piezoelectric transducer is vertically suspended to a support and that a force, F, is acting on its end. This force creates a variation in the length l of the sensor, Δl.

The related applied stress on the transducer, $X=Y\frac{\Delta l}{l}$, is given by the force divided by the cross-sectional area, csa = wt (where w is the width and t is the thickness), so that:

(2) $$Y\frac{\Delta l}{l}=\frac{F}{\text{wt}}$$

In this way, it is possible to write the equation for the generated electrical energy, $U_{e}$, in terms of the applied force [30]:

(3) $$U_{e}=\frac{1}{2}\frac{\varepsilon_{0}\varepsilon_{r}\text{wl}}{t}V^{2}=\frac{1}{2\varepsilon_{0}\varepsilon_{r}}\frac{l}{\text{wt}}d_{31}^{2}F^{2}$$

### 3.2. The Chosen Human Body Movements

Seven body joints were chosen in order to harvest energy from body movements. These joints were chosen in order to measure the most common human body rotations: neck, shoulder, elbow, wrist, hip, knee and ankle are the joints around which the transducers were placed.

The angle values were manually measured by a goniometer, for each range of motion of the performed tests. These results were accepted on the basis of the results presented in the work of Reese and Bandy [31]. In Table 2, the body joints, joint movements, range of motion and the frequencies of motion are summarized.

### 3.3. The Chosen Human Body Activity

Five common locomotion activities were chosen to find out if the two piezoelectric transducers were efficient enough to harvest energy from the body movement in practice. The mentioned activities were walking, going down and up the stairs, jogging and running. The body joints chosen for the comparison of the two piezoelectric transducers were a subset of the aforementioned ones: elbow, ankle, knee, shoulder and hip. The flexion-extension of the wrist and neck were excluded from the measurements because these movements were deemed as minor in terms of exerted forces during the chosen locomotion activities. In Table 3, the activities and their corresponding frequencies are summarized.

### 3.4. Elastic Cotton Fabric for a Tight Suit

Piezoelectric transducers must be placed directly onto the skin to transduce the movements of the joints in the most appropriate way. Thus, a tight elastic cotton suit was made. Elastic cotton ensures proper adjacency to the body and comfort, and it is easy to clean.

**Figure 2:**
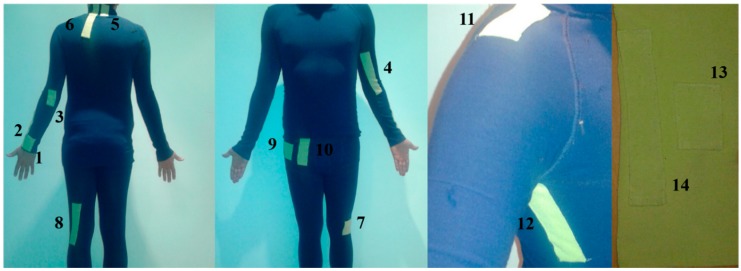
The blue suit with the green pockets and a green belt (please see Table 4 for denotation).

The piezoelectric transducers were placed in the pockets of the suit, around each chosen joint. Figure 2 shows the blue suit with the green pockets, and a green belt which was used to capture ankle rotations.

The positions of the transducers on the suit were chosen according to the value of their folding parameter, the minimum radius curvature. LDT4-028k is more flexible than P-876.A12, and therefore the former one was placed in the inner parts of the suit where the joints are at the maximum bending angle. The inner parts of the suit relate to the inner parts of the elbow, knee and shoulder. Conversely, P-876.A12 transducers were placed on the outer parts of those joints. The positions of transducers were the same for the measurements of the wrist, neck, ankle and hip joints. Table 4 shows the reference positions for the transducers referring to the numbers in Figure 2.

## 4. Experimental Section

### 4.1. Preliminary Measurements

**Figure 3:**
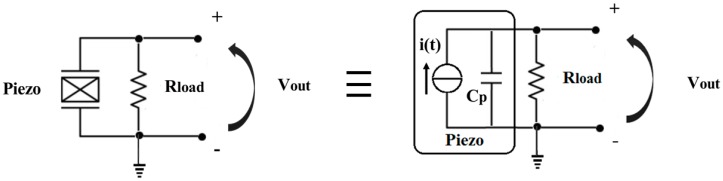
Measurement circuit.

To find out the voltage output, the following measurement circuit (Figure 3) was used, and the values of the voltage output were measured and acquired by the NI USB-6210 data acquisition system (DAQ), National Instruments. A voltage divider circuit was used as the input stage of the NI USB-6210 DAQ in order to avoid the problem of saturation given by the range of voltage values at the input stage of the DAQ.

The voltage output for the circuit in Figure 3 is given by the following Equation (4) [32]:

(4) $$V_{\text{out}}=I\frac{R_{\text{load}}}{\sqrt{1+{\left(2{\pi \text{fC}}_{p}R_{\text{load}}\right)}^{2}}}$$

The power values, based on a fixed value of the resistive load, were calculated. In order to optimize the power output, the ranges of fixed values of the resistors were carefully chosen based on the transducers datasheets [33,34] and on the motion frequency of the joint movements, so that the following relation is achieved:

(5) $$\frac{{\text{dP}}_{\text{out}}}{{\text{dR}}_{\text{load}}}=\frac{d\left(\frac{V_{\text{out}}^{2}}{R_{\text{load}}}\right)}{{\text{dR}}_{\text{load}}}=0\Leftrightarrow R_{\text{load}}=\frac{1}{2{\pi \text{fC}}_{p}}$$

The range values of the resistor loads are shown in Table 5.

To ensure that the P-876.A12 transducer could be bent properly, perfectly following the whole movement of the joints, it was necessary to place an extra elastic band over the suit, to make the transducers adhere to the body part during movement. Conversely, the LDT4-028k transducers did not need any additional support to secure their placement, due to their higher flexibility and ability to follow the joint movements.

To obtain the root mean square values of the voltages, currents and powers, the measured values of the peak-to-peak voltage output were used in the following Equation (6).

(6) $$V_{\text{rms}}=\frac{V_{\text{out}}}{2\sqrt{2}}\Rightarrow I_{\text{rms}}=\frac{V_{\text{rms}}}{R_{\text{fixed}}}\Rightarrow P_{\text{rms}}=V_{\text{rms}}I_{\text{rms}}$$

**Figure 4:**
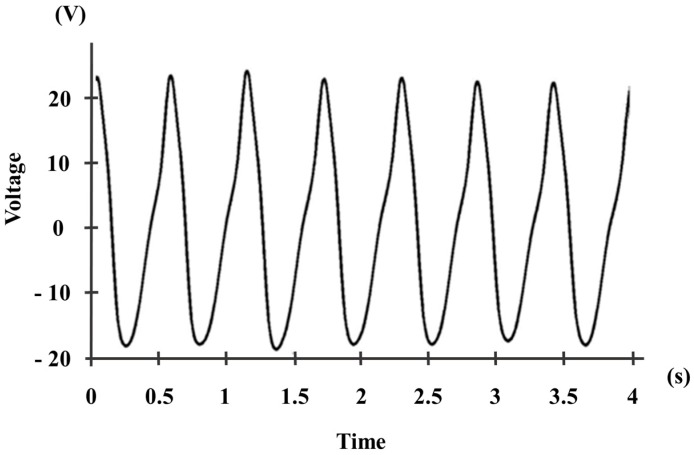
Sample of the signal of the voltage output measured by using the LDT4-028k transducer placed on the elbow joint for the movement of flexion-extension. The calculated RMS value corresponds to 13.78 V, slightly less than the value that could have been obtained if Equation (6), for sinusoidal waves, had been applied (14.62 V).

Equation (6) was used to find the RMS values from each performed movement repetition, shown in Table 2. In the case of these movements, the nature of the angular movements could be roughly approximated to Equation (6), even if it deviates from a pure sinusoidal tone, as it is shown in Figure 4.

Three healthy male volunteers (age: 34 ± 5 years; body weight: 76 ± 4 kg; height: 175 ± 5 cm) were recruited to perform the joint movements listed in Table 2. Each joint movement was repeated five times, and the measuring time for each test was around ten seconds.

**Figure 5:**
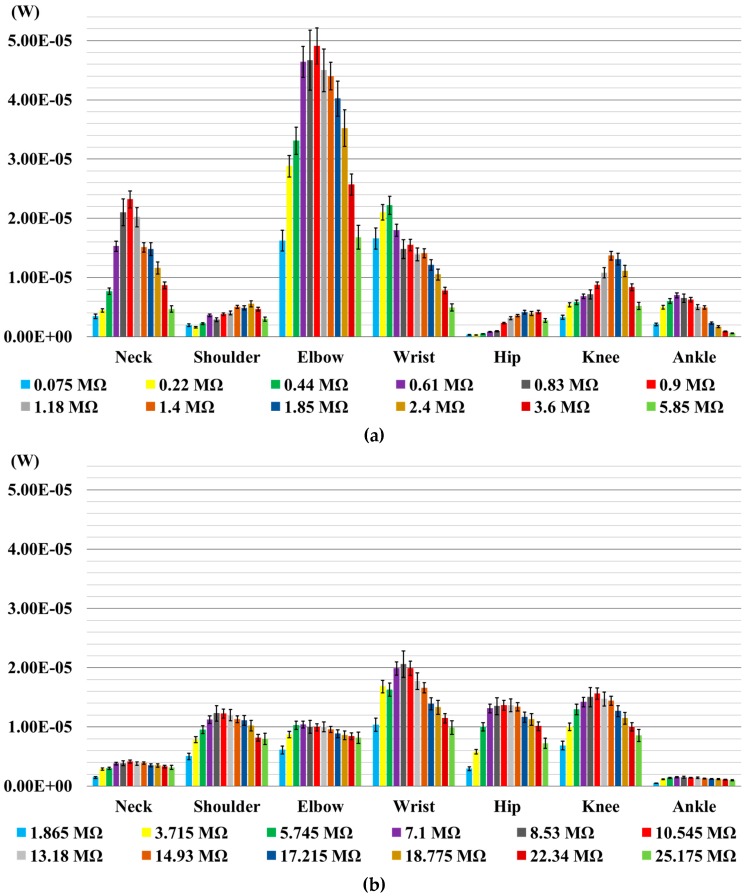
The calculated mean values of the power output for each value of the load resistor. Error bars represent standard deviations. (**a**) P-876.A12; (**b**) LDT4-028k.

Figure 5 shows output RMS power values coming from the body joints for each value of the resistive load. Both transducers produce a signal output proportional to the folding movement and frequency of the performed test. To obtain the maximum value of the power output, the most important parameter is the proper positioning of the transducer on the suit close to the skin, so that it can be bent as much as possible. The inherent different nature of the transducers makes it difficult to use the same exact location for both sensors, since the rigidity of the P-876.A12 transducer makes it necessary to provide the user with a sustaining structure and a different placement as compared to the LDT4-028k transducer. This consideration makes it impossible to directly compare the power output of transducers, but since the focus of the work is on the applicability of the transducers in real-life conditions, the comparison between transducers needs to include the positioning choice, as well.

In the P-876.A12 transducer diagram (Figure 5a), the power outputs for the elbow joint are higher than those obtained for the other joints. In the graph of the LDT4-028k transducer (Figure 5b), the highest power output values are those related to wrist, knee and hip joints. For both transducers, the values of the power output increase with the increase of R_load_ to reach a maximum value, and then, they gradually decrease. Comparing the graphs in Figure 5a,b, the maximum values of the power output for P-876.A12 on the elbow (49.1 µW), ankle (4.94 µW), wrist (22.2 µW) and neck (7.68 µW) joints are higher than those obtained by LDT4-028k on the same joints. Conversely, the maximum values of the power output for LDT4-028k on the shoulder (12.3 µW), knee (15.9 µW) and hip (15.0 µW) joints are higher than those appearing for P-876.A12 placed on the same joints.

### 4.2. Measurements of the Power Output during Locomotion Activities

Five common activities were chosen to test the two piezoelectric transducers. The common activities were walking, walking down and up the stairs, jogging and running, and the chosen joints for the tests were the shoulder, elbow, hip, knee and ankle. For each activity, voltages were measured and acquired by the NI USB-6210 DAQ, National Instruments, while current and power values, based on a fixed value of the resistive load, were calculated using MATLAB software. A voltage divider circuit was used as the input stage of the NI USB-6210 DAQ to avoid saturation given by the range of voltage values at the input stage of the DAQ. The fixed values of the resistive load, based on the previous measurements (Figure 5), were carefully chosen for each joint, and they are shown in Table 6. These values were obtained through interpolation and approximation of the discrete values tested in the first experimentation.

The tests were repeated for each reported resistance value, and thus, the values of the power output were obtained in statistical terms as average values across multiple repetitions, to take into account the inherent variability of having different participant population samples repeating the mentioned activities.

Three healthy male volunteers (age: 34 ± 5 year; body weight: 76 ± 4 kg; height: 175 ± 5 cm) were recruited to perform the activities listed in Table 3. Each task was repeated four times. The measuring time of walking and walking down and up the stairs was one minute for each test, while the measuring time of the jogging activity was twenty seconds and for the running activity eight seconds.

Also for these tests, the inherent different nature of the transducers makes it difficult to use the same exact location for both sensors, since the rigidity of the P-876.A12 transducer makes it necessary to provide the user with a sustaining structure and a different placement as compared to the LDT4-028k transducer.

**Figure 6:**
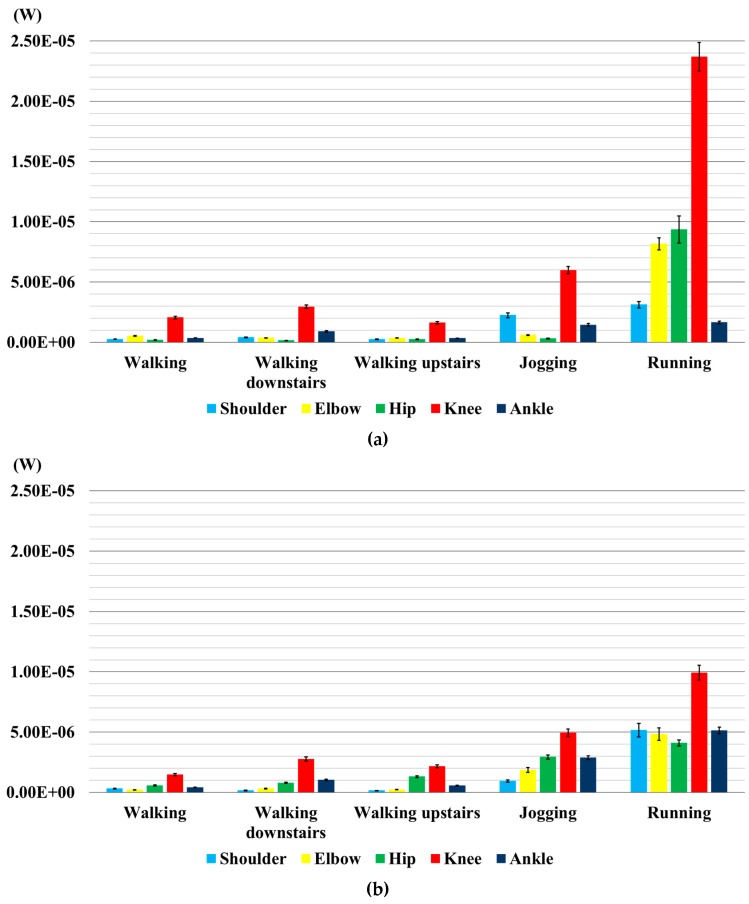
Comparison of the calculated grand mean values of power output on each joint for each activity. Error bars represent standard deviations. (**a**) P-876.A12; (**b**) LDT4-028k.

Figure 6 shows the measurements of the RMS power output values of the body joints, for each performed activity. Locomotion activities, such as walking and walking up stairs, produce the lowest values of the power output for both transducers, while running produces the highest values for both of them, followed by power output values of jogging and walking down stairs.

As it can be clearly seen in Figure 6a, walking, walking up stairs and walking down stairs, for P-876.A12, led to very similar power output. Out of these three activities, the knee joint produces the highest values of power output (2.21 µW) followed by the ankle (0.54 µW), elbow (0.41 µW), shoulder (0.31 µW) and hip (0.20 µW) joints. As for the jogging activity, the knee joint produces the highest value of power output (5.98 µW) followed by the shoulder (2.24 µW), ankle (1.45 µW), elbow (0.60 µW) and hip (0.31 µW) joints. As for running, the knee joint produces the highest value of the power output (23.70 µW) followed by hip (9.37 µW), elbow (8.16 µW), shoulder (3.12 µW) and ankle (1.65 µW) joints.

Similar considerations apply to the LDT4-028k transducer (Figure 6b), though with lower absolute values. In all activities, except running and jogging, the knee joint produces the highest value of power output (1.90 µW) followed by the hip (0.95 µW), ankle (0.67 µW), elbow (0.25 µW) and shoulder (0.21 µW) joints. As for jogging, the knee joint produces the highest value of power output (3.71 µW) followed by the ankle (2.88 µW), hip (2.43 µW), elbow (1.86 µW) and shoulder (4.46 µW) joints. As for running, the knee joint produces the highest value of power output (8.83 µW) followed by the shoulder (5.16 µW), ankle (5.13 µW), elbow (4.83 µW) and hip (4.46 µW) joints.

Table 7 breaks down the comparison between P-876.A12 (PZT) and LDT4-028k (PVDF) considering all the locomotion activities and all the joints, highlighting which transducer performed best for each activity and joint.

During walking, the values of the power output measured at each joint were very similar for both transducers: elbow (+0.31 µW) and knee (+0.47 µW) joints provide better results with P-876.A12, while the hip joint (+0.66 µW) is better with LDT4-028k.

Similar considerations apply to the case of walking down stairs, with slightly better results on the shoulder joint (+0.26 µW) for P-876.A12 and better on the hip joint (+0.52 µW) for LDT4-028k.

In the case of walking up stairs, LDT4-028k provides clearly better results than P-876.A12 at the hip joint (+1.08 µW). As for the jogging activity, P-876.A12 provides better results both at the shoulder (+1.30 µW) and at the knee (+2.27 µW) joints, while LDT4-028k resulted in being better for the elbow (+1.26 µW), hip (+2.12 µW) and ankle (+1.43 µW) joints. Finally, in the case of running, P-876.A12 performed better than LDT4-028k at the elbow (+3.33 µW), hip (+4.91 µW) and knee (+14.87 µW) joints, while the opposite applies to the shoulder (−2.04 µW) and ankle (−3.48 µW) joints.

**Figure 7:**
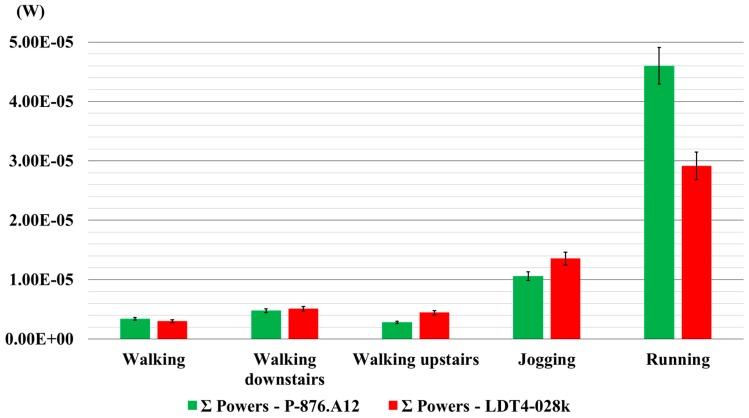
The sums of the mean values of the calculated power output on each joint for the activity performed. Error bars represent the standard deviation.

The graph in Figure 7 shows the results of the overall comparison between the transducers, by considering the sum of the power output values measured at each joint for each locomotion activity. From the overall comparison, transducers are similar in terms of power generation for both walking and walking down stairs; when walking upstairs and jogging, LDT4-028k is more efficient than P-876.A12. Finally, during running, even if both transducers are appropriate for use as energy harvesters, the PZT transducer performed better than PVDF. The numerical values are summarized in Table 8.

Table 9 breaks down the power generated by the ones that come from the upper body joints and the ones coming from the lower limb joints. The power contribution from the upper body joints is almost negligible for the three walking activities, while it can be considered as a significant source of power generation for both jogging and running.

## 5. Discussion

As it can be clearly seen from the tests shown in the previous section, the power output measured at each joint is sufficient enough to consider both transducers suitable to be used as energy harvesters for BMEH applications.

When comparing the performed tests, it resulted that soft PZT technology is more efficient than PVDF in terms of generated power output; however, PVDF technology is more comfortable in terms of user wearability.

The results obtained in this work are in line with the results of the current wearable fabrics for BMEH found in the present scientific literature and reported below as follows: Zhang *et al.* [22] developed a fabric nanogenerator able to produce 10.02 nW when it is attached on an elbow pad and bent by human arms. Yang and Yun [35] prepared three fabrics in the form of a band for wearing on elbow joint, measuring 0.21 mW for a bending velocity of 5 rad/s. Dhakar *et al.* [36] presented a triboelectric nanogenerator able to generate voltages up to 90 V with a mild finger touch, and Yang *et al.* [37] developed a flexible triboelectric nanogenerator for energy harvesting from various types of mechanical motions, able to deliver an open-circuit voltage of 700 V and a short-circuit current of 75 µA. Pu *et al*. [38] developed a textile triboelectric nanogenerator able to generate 20, 2 and 0.8 µA rectified output currents by foot pressing, arm swinging and elbow bending, respectively. Yun *et al.* [39] presented a very flexible harvester design that can elastically stretch to 1.6-times its normal length, allowing it to be used on a large range of motion body areas. Li *et al.* [40] developed a power shirt based on triboelectrification and the electrostatic induction effect, able to achieve a maximum peak power density of 4.65 µW/cm^2^, and Wu *et al.* [41] produced a wearable nanogenerator able to produce 6 V of output voltage and 45 nA of output current.

The results of the power output harvested from the transducers in this paper represent the power output generated by the five locomotion activities and, thus, may represent an added value to the results found in the current scientific literature, which represent values of the power output generated only from individual body movements.

## 6. Conclusions

In this paper, two piezoelectric transducers were placed inside a tight wearable suit in proximity to the main human body joints, neck, shoulder, elbow, wrist, hip, knee and ankle, respectively, in order to harvest energy generated by common body movements in the form of casual walking, walking down and up stairs, jogging and running.

In order to work at its best, it is very important for a BMEH system using the piezoelectric transducers to ensure as close contact as possible between the transducers and the skin; therefore, a special manufactured body suit was produced to be worn during the activities executed in this work.

When examining the user wearability of the two transducers, PVDF technology is more adequate than soft PZT, because the value of its folding parameter is higher: as a result, it is more comfortable for the user and can better adhere to body movements.

When examining the power output measured during the five common activities, the values of the power output are in the range of 2–46 µW/cm^3^ for a single transducer for a joint, while using both transducers for a joint, the values of the power output are in the range of 6 µW–74 µW, thus confirming the possibility to include these harvesters into more general systems for long-term monitoring.

In order to continue the work on the development of a system for BMEH, PVDF material has produced the best results. The soft PZT technology produced higher values of power output, but its lack of comfort makes it difficult to be worn in long-term activities, to adequately follow the body segment movements.

## Figures and Tables

**Table 1:** Properties of the P-876.A12 and LDT4-028k transducers.

Properties	P-876.A12	LDT4-028k
Density, ρ (g/cm^3^)	7.8	1.8
Curié temperature, T_c_ (°C)	350	100
Relative permittivity, ε_r_	1650–1750	12–13
Dielectric loss factor, σ	0.02	0.02
Coupling factor, k_t_	0.47	0.14
Coupling factor, k_31_	0.35	0.12
Coupling factor, k_33_	0.69	-
Piezo charge coefficient, |d_31_| (10^−12^ C/N)	180	23
Piezo charge coefficient, |d_33_| (10^−12^ C/N)	400	33
Piezo voltage coefficient,|g_31_| (10^−3^ V m/N)	11.3	216
Piezo voltage coefficient,|g_33_| (10^−3^ V m/N)	25	330
Young’s modulus, Y (10^9^ N/m^2^)	55.5	3.1
Minimum radius curvature, r_c_ (mm)	20	5
Active vol. dimensions (l × w × t) (mm)	(50 × 30 × 0.2)	(156 × 19 × 0.028)
Electrical capacitance, C (10^−9^ F)	90	12

**Table 2:** Body joints, body joint movements, range of motion [31] and frequency of tests.

Body Joint	Joint Movement	Range of Motion (°)	Motion Frequency (Hz)
Neck	flexion-extension	58 ± 12	1.40± 0.10
Shoulder	adduction-abduction	140 ±9	1.10 ± 0.10
Elbow	flexion-extension	141 ± 8	1.25 ± 0.10
Wrist	flexion-extension	129 ± 14	1.90 ± 0.10
Hip	flexion-extension	138 ± 21	1.05 ± 0.10
Knee	flexion-extension	135 ± 11	1.15 ± 0.10
Ankle	plantar-dorsiflexion	66 ± 5	1.75 ± 0.10

**Table 3:** Summary of the common activities and their frequencies.

Common Activity	Frequency of the Activity (Hz)
Walking	1.15 ± 0.10
Walking downstairs	1.65 ± 0.10
Walking upstairs	1.05 ± 0.10
Jogging	1.60 ± 0.10
Running	2.10 ± 0.10

**Table 4:** Positions of the transducers on the suit and on the belt.

Numbers in Figure 2	Joint, Transducer	Reference Position for the Measurements
1	Wrist, P-876.A12	Top of wrist
2	Wrist, LDT4-028k	Top of wrist
3	Elbow, P-876.A12	Outer elbow
4	Elbow, LDT4-028k	Inner elbow
5	Neck, P-876.A12	Back of the neck
6	Neck, LDT4-028k	Back of the neck
7	Knee, P-876.A12	Outer knee
8	Knee, LDT4-028k	Inner knee
9	Hip, P-876.A12	Front hip
10	Hip, LDT4-028k	Front hip
11	Shoulder, P-876.A12	Outer shoulder
12	Shoulder, LDT4-028k	Inner shoulder
13	Ankle, P-876.A12	The ankle plantar
14	Ankle, LDT4-028k	The ankle plantar

**Table 5:** The chosen fixed values of the resistors.

R_load_ → P-876.A12 Transducer	R_load_ → LDT4-028k Transducer
0.075 MΩ	1.865 MΩ
0.220 MΩ	3.715 MΩ
0.440 MΩ	5.745 MΩ
0.610 MΩ	7.100 MΩ
0.830 MΩ	8.530 MΩ
0.900 MΩ	10.545 MΩ
1.180 MΩ	13.180 MΩ
1.400 MΩ	14.930 MΩ
1.850 MΩ	17.215 MΩ
2.400 MΩ	18.775 MΩ
3.600 MΩ	22.340 MΩ
5.850 MΩ	25.175 MΩ

**Table 6:** The fixed values of the resistors for each joint.

Joint	R_load_ → P-876.A12	R_load_ → LDT4-028k
Shoulder	2.000 MΩ	8.880 MΩ
Elbow	0.952 MΩ	7.880 MΩ
Hip	2.000 MΩ	10.565 MΩ
Knee	1.430 MΩ	10.565 MΩ
Ankle	0.952 MΩ	7.080 MΩ

**Table 7:** Comparison between the two transducers based on the five locomotion activities: lead zirconate titanate (PZT) and polyvinylidene fluoride (PVDF).

Joint	Walking		Walking down Stairs		Walking up Stairs		Jogging		Running	
	PZT	PVDF	PZT	PVDF	PZT	PVDF	PZT	PVDF	PZT	PVDF
Shoulder	=	=	+	-	=	=	+	-	-	+
Elbow	+	-	=	=	=	=	-	+	+	-
Hip	-	+	-	+	-	+	-	+	+	-
Knee	+	-	=	=	=	=	+	-	+	-
Ankle	=	=	=	=	=	=	-	+	-	+

Differences in power harvesting between transducers: the symbol “+” represents the transducer with the highest value of power output, and conversely, the symbol “-” represents the transducer with the lowest value of power output. The symbol “=” represents transducers of very similar values of power output.

**Table 8:** Results of the sum of the power grand mean values measured at all of the joints for each activity.

Activity	∑ Power, P-876.A12	∑ Power, LDT4-028k
Walking	3.39 µW	3.38 µW
Walking downstairs	4.79 µW	4.38 µW
Walking upstairs	2.81 µW	4.18 µW
Jogging	10.60 µW	11.81 µW
Running	46.00 µW	28.41 µW

**Table 9:** Results shown in Table 8 divided by joint position: upper body and lower body.

Activity	∑ Power, P-876.A12		∑ Power, LDT4-028k	
	Upper Body	Lower Body	Upper Body	Lower Body
Walking	0.78 µW	2.61 µW	0.52 µW	2.86 µW
Walking downstairs	0.78 µW	4.01 µW	0.47 µW	3.91 µW
Walking upstairs	0.60 µW	2.21 µW	0.39 µW	3.79 µW
Jogging	2.84 µW	7.74 µW	2.81 µW	9.00 µW
Running	11.28 µW	34.72 µW	9.99 µW	18.42 µW
